# Hypoxia-inducible factors regulate pluripotency factor expression by ZNF217- and ALKBH5-mediated modulation of RNA methylation in breast cancer cells

**DOI:** 10.18632/oncotarget.11743

**Published:** 2016-08-31

**Authors:** Chuanzhao Zhang, Wanqing Iris Zhi, Haiquan Lu, Debangshu Samanta, Ivan Chen, Edward Gabrielson, Gregg L. Semenza

**Affiliations:** ^1^ Institute for Cell Engineering, Johns Hopkins University School of Medicine, Baltimore, MD, USA; ^2^ Department of General Surgery, Guangdong General Hospital, Guangdong Academy of Medical Sciences, Guangzhou, China; ^3^ Department of Oncology, Johns Hopkins University School of Medicine, Baltimore, MD, USA; ^4^ Department of Pathology, Johns Hopkins University School of Medicine, Baltimore, MD, USA; ^5^ Department of Pediatrics, Johns Hopkins University School of Medicine, Baltimore, MD, USA; ^6^ Department of Medicine, Johns Hopkins University School of Medicine, Baltimore, MD, USA; ^7^ Department of Radiation Oncology, Johns Hopkins University School of Medicine, Baltimore, MD, USA; ^8^ Department of Biological Chemistry, Johns Hopkins University School of Medicine, Baltimore, MD, USA; ^9^ McKusick-Nathans Institute of Genetic Medicine, Johns Hopkins University School of Medicine, Baltimore, MD, USA

**Keywords:** breast cancer stem cells, hypoxia, metastasis, N^6^-methyladenosine, pluripotency factors

## Abstract

Exposure of breast cancer cells to hypoxia increases the percentage of breast cancer stem cells (BCSCs), which are required for tumor initiation and metastasis, and this response is dependent on the activity of hypoxia-inducible factors (HIFs). We previously reported that exposure of breast cancer cells to hypoxia induces the ALKBH5-mediated demethylation of N^6^-methyladenosine (m^6^A) in NANOG mRNA leading to increased expression of NANOG, which is a pluripotency factor that promotes BCSC specification. Here we report that exposure of breast cancer cells to hypoxia also induces ZNF217-dependent inhibition of m^6^A methylation of mRNAs encoding NANOG and KLF4, which is another pluripotency factor that mediates BCSC specification. Although hypoxia induced the BCSC phenotype in all breast-cancer cell lines analyzed, it did so through variable induction of pluripotency factors and ALKBH5 or ZNF217. However, in every breast cancer line, the hypoxic induction of pluripotency factor and ALKBH5 or ZNF217 expression was HIF-dependent. Immunohistochemistry revealed that expression of HIF-1α and ALKBH5 was concordant in all human breast cancer biopsies analyzed. ALKBH5 knockdown in MDA-MB-231 breast cancer cells significantly decreased metastasis from breast to lungs in immunodeficient mice. Thus, HIFs stimulate pluripotency factor expression and BCSC specification by negative regulation of RNA methylation.

## INTRODUCTION

Cancer stem cells are a small subpopulation of cells within various tumor types, including breast cancers, which have the dual properties of self-renewal and differentiation by giving rise to both daughter cancer stem cells and bulk (non-stem) cancer cells [1, 2]. Cancer stem cells play an essential role in tumor initiation and progression [3]. Breast cancer stem cells (BCSCs) are resistant to chemotherapy and may constitute the residual cell population that is the source of recurrent and metastatic tumors that result in patient mortality [4, 5]. Indeed, treatment of breast cancer cells with carboplatin, gemcitabine, or paclitaxel *in vitro* or *in vivo* increases the percentage of BCSCs among the surviving cells [6-8]. Thus, delineation of the molecular mechanisms that regulate the BCSC phenotype is needed in order to design more effective therapies.

The BCSC phenotype is specified and maintained by the expression of core pluripotency factors, including octamer-binding transcription factor 4 (OCT4), Kruppel-like factor 4 (KLF4), SRY-box 2 (SOX2), and NANOG [9-12]. In recent studies, we found that hypoxia-inducible factors (HIFs) mediated increased NANOG, SOX2, and OCT4 expression in human breast cancer cells in response to chemotherapy or hypoxia [8, 13]. In several breast cancer cell lines, hypoxia induced the HIF-dependent expression of AlkB homolog 5 (ALKBH5) [13, 14], which is an enzyme that removes *N*^6^-methyl groups from adenosine residues in RNA. [15] ALKBH5-mediated demethylation of NANOG mRNA increased its stability, leading to increased NANOG protein expression, and ALKBH5 was required for hypoxic induction of the BCSC phenotype [13], as previously demonstrated for HIF-1α [16, 17].

Hypoxia induced ALKBH5 expression in MCF-7, MDA-MB-231, and SUM-159 breast cancer cells, but not in HCC-1954, SUM-149, T47D, or ZR75.1 cells [13], illustrating the heterogeneous nature of the transcriptional response to hypoxia. These results suggested either that *N*^6^-methyladenosine (m^6^A) levels in NANOG (and other mRNAs) were not oxygen-regulated in these non-responding cell lines, or that they were regulated by an alternative mechanism, such as increased expression of fat mass and obesity associated protein (FTO), which is the other known m^6^A demethylase, or by inhibition of the m^6^A methyltransferase complex, which is comprised of the proteins methyltransferase-like 3 (METTL3), METTL14, and Wilms tumor 1 associated protein [18, 19]. Our previous study also did not investigate whether, in addition to NANOG, the expression of other pluripotency factors is regulated by m^6^A demethylation of mRNA in hypoxic breast cancer cells.

The *ZNF217* gene on human chromosome 20q13.2 encodes a transcription factor that is overexpressed in breast cancer [20]. Increased ZNF217 expression is correlated with patient mortality in breast cancer and glioma [21, 22]. A recent study showed that in embryonic stem (ES) cells Zfp217, which is the mouse homolog of ZNF217, inhibited m^6^A modification of NANOG, KLF4 and SOX2 mRNA by sequestering METTL3 [23]. Interestingly, ZNF217 expression was induced by hypoxia in a HIF-dependent manner in glioma cells [21]. Based these data, we hypothesized that ZNF217 may also inhibit m^6^A modification of pluripotency factor mRNAs in hypoxic breast cancer cells to promote the BCSC phenotype.

In the current study we have comprehensively analyzed seven representative human breast cancer cell lines to determine the effect of hypoxia on the percentage of BCSCs and on the expression of pluripotency factors (NANOG, KLF4 and SOX2), m^6^A demethylases (ALKBH5 and FTO), and an m^6^A methyltransferase inhibitor (ZNF217). We have also analyzed the effect of ALKBH5 or ZNF217 loss of function on the BCSC phenotype and breast cancer metastasis.

## RESULTS

### Hypoxia induces BCSC enrichment

**Figure 1:**
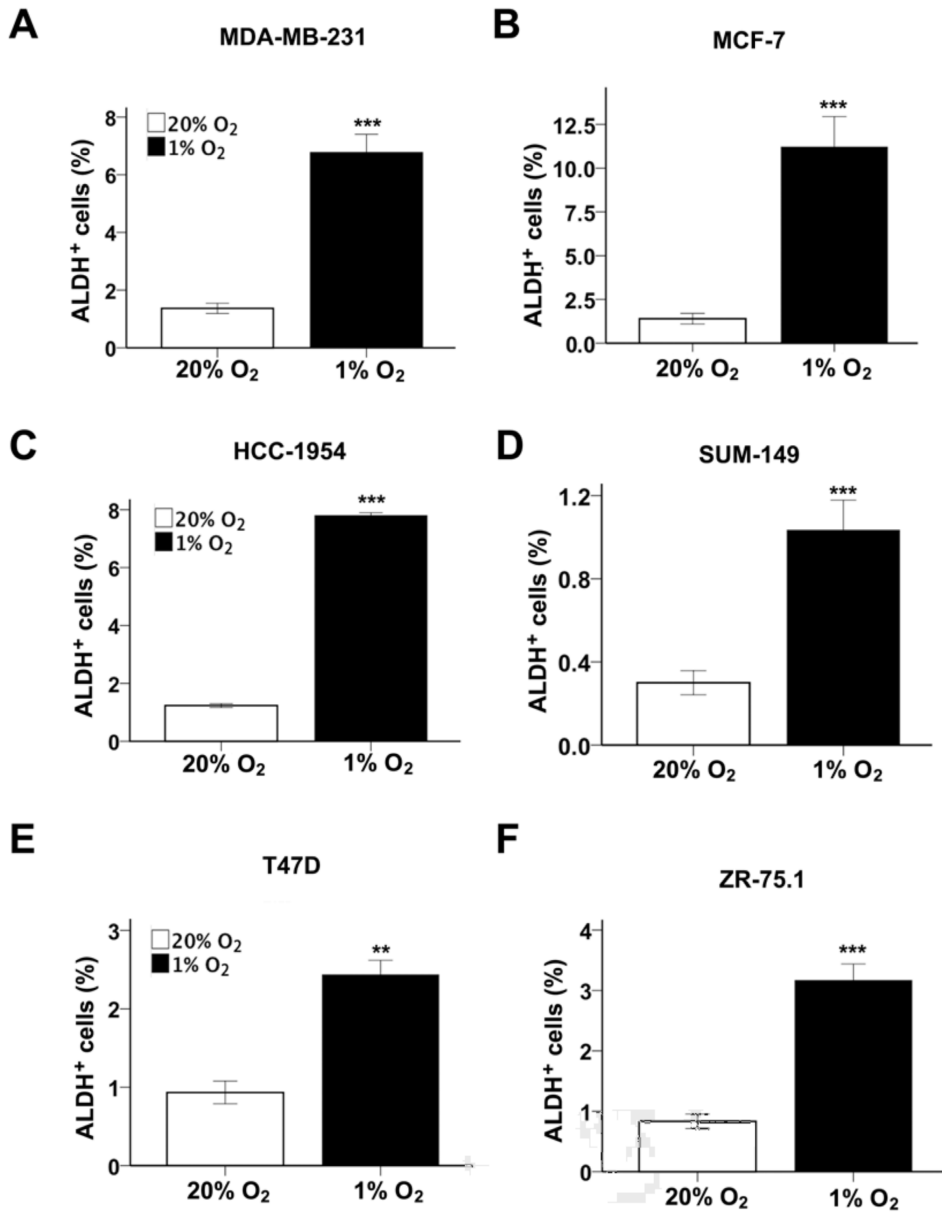
Hypoxia induces BCSC enrichment **A-F.** The following breast cancer cell lines were exposed to 20% or 1% O_2_ for 72 h and the percentage of cells expressing aldehyde dehydrogenase (ALDH^+^) was determined (mean ± SEM; *n* = 3): MDA-MB-231 (A), MCF- 7 (B), HCC-1954 (C), SUM-149 (D), T47D (E), and ZR-75.1 (F). **P < 0.01, ***P < 0.001 *vs*. same cell line at 20% O_2_ by Student's *t* test.

Human breast cancers are classified clinically based on their expression of the estrogen receptor (ER), progesterone receptor (PR), and human epidermal growth factor receptor 2 (HER2). We studied a panel of seven breast cancer cell lines derived from ER^+^ (ZR75.1), ER^+^PR^+^ (MCF-7 and T47D), HER2^+^ (HCC-1954), and triple-negative (MDA-MB-231, SUM-149, and SUM-159) breast cancers [24]. We first investigated the effect of hypoxia on BCSCs by analyzing aldehyde dehydrogenase 1 (ALDH) activity, which identifies a subpopulation of breast cancer cells that is enriched for tumor-initiating BCSCs [25]. We previously reported that exposure of SUM-159 cells to 1% O_2_increased the percentage of ALDH^+^ cells [26]. When the other six breast cancer cell lines were exposed to non-hypoxic (20% O_2_) or hypoxic (1% O_2_) conditions for 72 h, the percentage of ALDH^+^ cells was significantly increased under hypoxic conditions in all lines, with the induction ranging from 2.6-fold in T47D cells to 8-fold in MCF-7 cells (Figure 1). Thus, hypoxia serves as an important physiological stimulus, which is sufficient to promote BCSC enrichment in all breast cancer cell lines analyzed.

### Hypoxia induces pluripotency factor expression in a HIF-dependent and cell-specific manner

**Figure 2:**
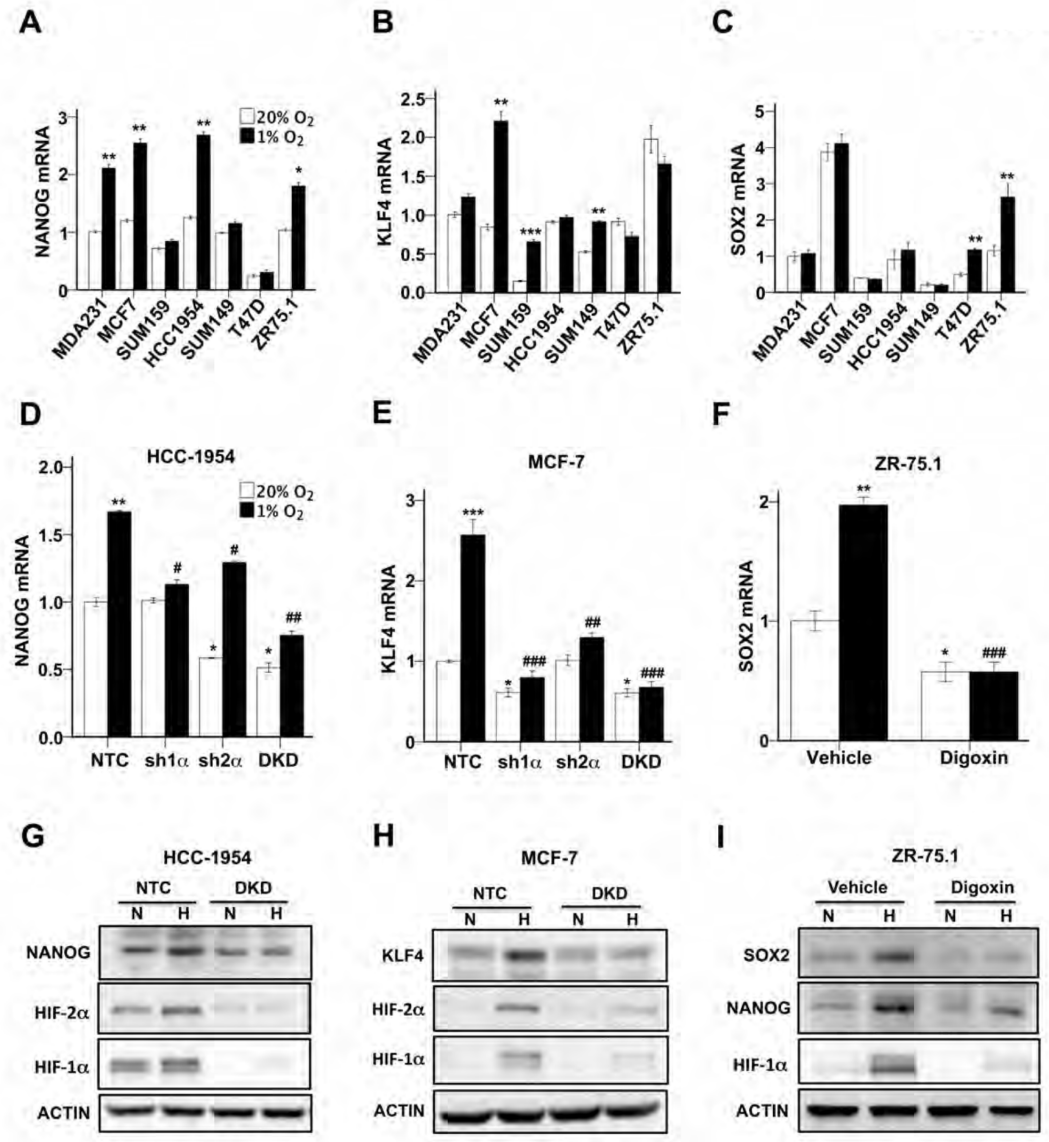
HIFs are required for hypoxia-induced expression of pluripotency factors **A-C.** Breast cancer cell lines were exposed to 20% or 1% O_2_ for 24 h and NANOG (A), KLF4 (B), and SOX2 (C) mRNA levels were determined by RT-qPCR, relative to 18S rRNA, and normalized to the mean value for MDA-MB-231 cells (MDA231) at 20% O_2_ (mean ± SEM; *n* = 3). *P < 0.05, **P < 0.01, ***P < 0.001 *vs*. same cell line at 20% O_2_ by Student's *t* test. **D** and **E.** HCC-1954 (D) and MCF-7 (E) subclones, which were stably transfected with an expression vector encoding a non-targeting control (NTC) shRNA, or vector encoding shRNA targeting HIF-1α (sh1α) or HIF-2α (sh2α), or vectors encoding shRNAs targeting both HIF-1α and HIF-2α (DKD), were exposed to 20% or 1% O_2_ for 24 h and RT-qPCR was performed to determine NANOG (D) or KLF4 (E) mRNA levels relative to 18S rRNA. The results were normalized to NTC at 20% O_2_ (mean ± SEM; *n* = 3). *P < 0.05, **P < 0.01, ***P < 0.001 *vs*. NTC at 20% O_2_; #*P* < 0.05, ##*P* < 0.01, ###*P* < 0.001 *vs*. NTC at 1% O_2_ by ANOVA. **F**. ZR75.1 cells treated with vehicle or digoxin (200 nM) were exposed to 20% or 1% O_2_ for 24 h and SOX2 mRNA was measured (mean ± SEM; *n* = 3). *P < 0.05, **P < 0.01 *vs*. NTC at 20% O2; ###*P* < 0.001 *vs*. NTC at 1% O2 by ANOVA. **G** and **H**. NTC and DKD subclones of HCC-1954 (G) and MCF-7 (H) were exposed to 20% or 1% O_2_ for 48 h, whole cell lysates were prepared, and immunoblot assays were performed to analyze HIF-1α, HIF-2α, NANOG and KLF4 protein expression. Actin was also analyzed as a loading control. **I.** ZR75.1 cells were treated with vehicle or digoxin (200 nM), exposed to 20% or 1% O_2_ for 48 h, and HIF-1α, NANOG and SOX2 immunoblot assays were performed.

We next investigated whether the expression of pluripotency factors, including NANOG, KLF4 and SOX2, was induced under hypoxic conditions. Breast cancer cells were exposed to 20% or 1% O_2_ for 24 h and RNA was extracted for reverse transcription (RT) and quantitative real-time PCR (qPCR). NANOG mRNA expression was induced by hypoxia in MDA-MB-231, MCF-7, HCC-1954 and ZR-75.1 cells (Figure 2A). KLF4 mRNA expression was induced by hypoxia in MCF-7, SUM-159 and SUM-149 cells (Figure 2B). SOX2 mRNA expression was induced by hypoxia in T47D and ZR-75.1 cells (Figure 2C). Taken together, the results indicate a remarkably heterogeneous response to hypoxia (Table 1). However, in each breast cancer cell line, the expression of at least one pluripotency factor was induced by hypoxia.

**Table 1:** Induction of BCSCs, pluripotency factors and m^6^A-regulating proteins by hypoxia

	BCSCs	Pluripotency factors			m^6^A regulating proteins		
	ALDH	NANOG	SOX2	KLF4	ALKBH5	ZNF217	FTO
**MCF-7**	+	+	−	+	+	+	−
**MDA-MB-231**	+	+	−	−	+	−	−
**SUM-159**	+	−	−	+	+	−	−
**HCC-1954**	+	+	−	−	−	+	−
**SUM-149**	+	−	−	+	−	−	−
**T47D**	+	−	+	−	−	−	−
**ZR75.1**	+	+	+	−	−	+	−

Induction of mRNA expression (1% vs. 20% O_2_): +, > 1.5-fold increase; -, < 1.5-fold increase.

We next investigated whether the hypoxia-inducible expression of NANOG, KLF4, and SOX2 was HIF-dependent. In previous studies, we stably transfected MCF-7 and HCC-1954 cells with lentiviral expression vectors encoding a non-targeting control (NTC) short hairpin RNA (shRNA) or shRNA targeting HIF-1α (sh1α) or HIF-2α (sh2α) or both (double knockdown [DKD]) [17, 26]. Compared to the NTC subclone, NANOG and KLF4 mRNA levels were significantly decreased in the sh1α, sh2α, and DKD subclones of HCC-1954 (Figure 2D) and MCF-7 (Figure 2E) cells, respectively. We previously demonstrated that treatment of breast cancer cells with digoxin inhibits the hypoxic induction of HIF-1α protein and HIF target gene expression [27, 28]. Digoxin treatment significantly decreased SOX2 mRNA levels in hypoxic ZR75.1 cells (Figure 2F). Immunoblot assays revealed that expression of NANOG protein in HCC-1954 cells (Figure 2G) and KLF4 protein in MCF-7 cells (Figure 2H) was induced by hypoxia in NTC, but not in DKD, subclones. Digoxin treatment inhibited the expression of SOX2 and NANOG protein in hypoxic ZR-75.1 cells (Figure 2I). Taken together, these data indicate that hypoxia induces the expression of different pluripotency factors in different breast cancer cell lines but, in all cases, the induction is HIF-dependent.

### ZNF217 expression is induced by hypoxia in a HIF-dependent manner

**Figure 3:**
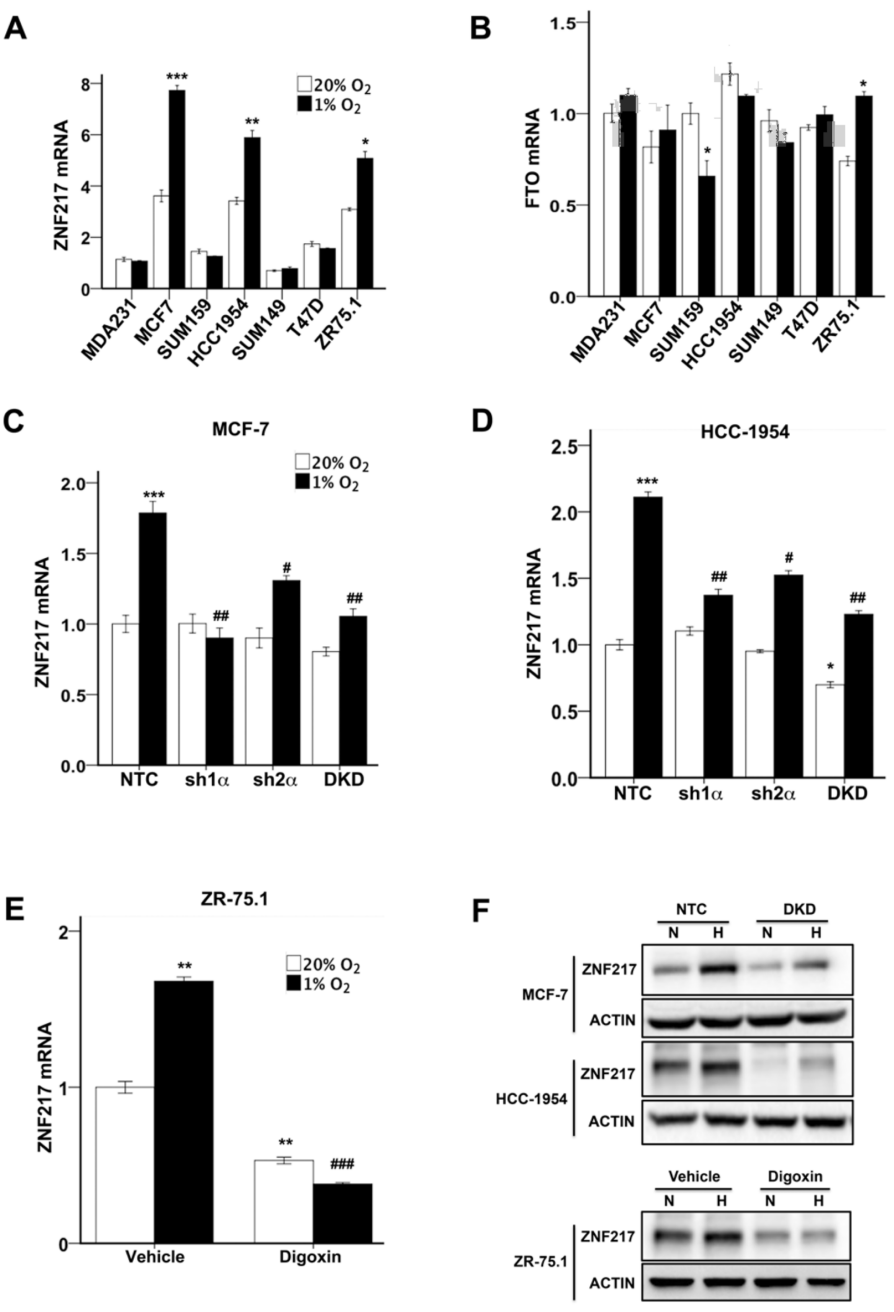
ZNF217, but not FTO, expression was induced by hypoxia in a HIF-dependent manner **A and B.** Breast cancer cell lines were exposed to 20% or 1% O_2_ for 24 h and ZNF217 (A) and FTO (B) mRNA levels were determined by RT-qPCR (mean ± SEM; *n* = 3). The data were normalized to the mean for the NTC-20% O_2_ condition. *P < 0.05, **P < 0.01, ***P < 0.001 *vs*. same cell line at 20% O_2_ by Student's *t* test (performed prior to normalization). **C** and **D**. MCF-7 (C) and HCC-1954 (D) subclones were exposed to 20% or 1% O_2_ for 24 h and ZNF217 mRNA levels were determined. *P < 0.05, ***P < 0.001 *vs*. NTC at 20% O_2_; #*P* < 0.05, ##*P* < 0.01 *vs*. NTC at 1% O_2_ by ANOVA. **E**. ZR75.1 cells treated with vehicle or digoxin (200 nM) were exposed to 20% or 1% O_2_ for 24 h and ZNF217 mRNA was measured (mean ± SEM; *n* = 3). **P < 0.01 *vs*. vehicle at 20% O_2_; ###*P* < 0.001 *vs*. vehicle at 1% O_2_ by ANOVA. **F**. MCF-7 and HCC-1954 subclones (upper panels), and ZR75.1 cells treated with vehicle or 200 nM digoxin (lower panels), were exposed to 20% or 1% O_2_ for 48 h and immunoblot assays were performed.

We previously demonstrated that hypoxia-induced m^6^A demethylation of NANOG mRNA by ALKBH5 positively regulated NANOG expression and the BCSC phenotype in MCF-7 and MDA-MB-231 breast cancer cells [13]. However, hypoxia increased the percentage of ALDH^+^ BCSCs (Figure 1) and expression of pluripotency factors (Figure 2) in all seven breast cancer cells analyzed, including four cell lines in which ALKBH5 expression was not induced by hypoxia (Table 1), suggesting that increased expression of ZNF217 or FTO might contribute to hypoxic induction of the BCSC phenotype. RT-qPCR analysis revealed that ZNF217 mRNA levels were significantly increased in MCF-7, HCC-1954, and ZR-75.1 cells under hypoxic conditions (Figure 3A). FTO mRNA levels were modestly increased (1.4 fold) in ZR-75.1 cells, decreased in SUM-159 cells, and unchanged in other breast cancer lines under hypoxic conditions (Figure 3B). Knockdown of HIF-1α, HIF-2α or both significantly inhibited the hypoxic induction of ZNF217 mRNA expression in both MCF-7 (Figure 3C) and HCC-1954 (Figure 3D) cells. Digoxin treatment of ZR-75.1 cells significantly decreased ZNF217 mRNA levels under both non-hypoxic and hypoxic conditions (Figure 3E). Immunoblot assays revealed that ZNF217 protein expression was induced by hypoxia in NTC, but not in DKD, subclones of HCC-1954 and MCF-7 cells (Figure 3F, upper panels). ZNF217 protein expression was decreased in digoxin-treated ZR75.1 cells under both non-hypoxic and hypoxic conditions (Figure 3F, lower panels). Taken together, the data in Figure 3 demonstrate that ZNF217 expression is induced by hypoxia in a HIF-dependent manner in a subset of human breast cancer cell lines.

### ZNF217 or ALKBH5 knockdown increases m^6^A RNA methylation and inhibits hypoxia-induced NANOG and KLF4 expression

**Figure 4:**
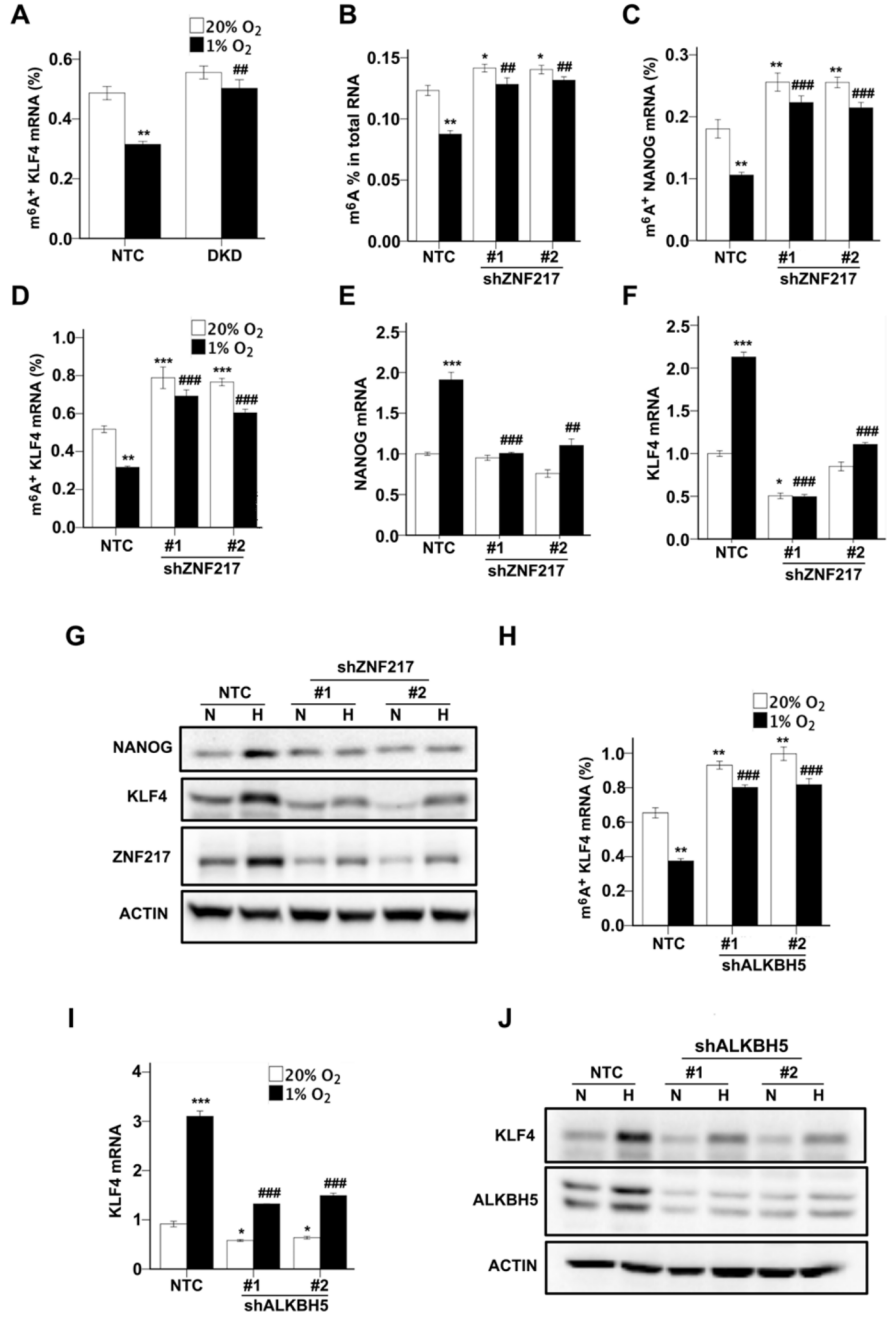
ZNF217 and ALKBH5 regulate NANOG and KLF4 expression *via* modulation of m6A levels **A.** MCF-7 subclones expressing a non-targeting control shRNA (NTC) or shRNAs targeting HIF-1α and HIF-2α (double knockdown [DKD]), were exposed to 20% or 1% O_2_ for 48 h. Immunoprecipitation of RNA with m6A antibody and RT-qPCR were performed to determine the percentage of KLF4 mRNA with methylation (m6A+) (mean ± SEM; *n* = 3). **P < 0.01 *vs*. NTC at 20% O_2_; ##*P* < 0.01 *vs*. NTC at 1% O_2_ by two-way ANOVA. **B.** MCF-7 subclones, which were stably transfected with lentiviral vector expressing NTC or either of two independent shRNAs targeting different nucleotide sequences in ZNF217, were exposed to 20% or 1% O_2_ for 24 h. Total RNA was extracted and m6A levels were determined as a percentage of all adenosine residues (mean ± SEM; *n* = 3). *P < 0.05, **P < 0.01 *vs*. NTC at 20%; ##*P* < 0.01 *vs*. NTC at 1% O_2_ by two-way ANOVA. **C** and **D.** MCF-7 subclones were exposed to 20% or 1% O_2_ for 48 h. The percentage of m6A+ NANOG (C) and KLF4 (D) mRNA was determined (mean ± SEM; *n* = 3). **P < 0.01 *vs*. NTC at 20% O_2_; ##*P* < 0.01, ###*P* < 0.001 *vs*. NTC at 1% O_2_ by two-way ANOVA. **E** and **F**. MCF-7 subclones were exposed to 20% or 1% O_2_ for 24 h. NANOG (E) and KLF4 (F) mRNA levels were determined. *P < 0.05, **P < 0.01, ***P < 0.001 *vs*. NTC at 20% O_2_; ##*P* < 0.01, ###*P* < 0.001 *vs*. NTC at 1% O_2_ by two-way ANOVA. **G.** MCF-7 subclones were exposed to 20% or 1% O_2_ for 48 h and immunoblot assays were performed. **H.** MCF-7 subclones were exposed to 20% or 1% O_2_ for 48 h. The percentage of m6A+ KLF4 mRNA was determined. *P < 0.05, **P < 0.01 *vs*. NTC at 20% O_2_; ##*P* < 0.01 *vs*. NTC at 1% O_2_ by two-way ANOVA. **I.** MCF-7 subclones were exposed to 20% or 1% O_2_ for 24 h and KLF4 mRNA levels were determined. *P < 0.05, ***P < 0.001 *vs*. NTC at 20% O_2_; ###*P* < 0.001 *vs*. NTC at 1% O_2_ by two-way ANOVA. **J.** MCF-7 subclones were exposed to 20% or 1% O_2_ for 48 h and immunoblot assays were performed.

We previously demonstrated that exposure of MCF-7 or MDA-MB-231 cells to hypoxia led to decreased m^6^A levels in total cellular RNA pools and decreased m^6^A modification of NANOG mRNA due to HIF-dependent ALKBH5 expression [13]. To investigate whether m^6^A modification of KLF4 mRNA was also regulated by hypoxia and HIFs, immunoprecipitation of m^6^A^+^ RNA from MCF-7 cells was performed, followed by RT-qPCR, which revealed that the levels of m^6^A^+^ KLF4 mRNA were significantly decreased under hypoxic conditions in the NTC, but not in the DKD, subclone (Figure 4A).

To test whether ZNF217 is required for the HIF-dependent decrease in m^6^A content of total RNA pools and of NANOG and KLF4 mRNA, we transfected MCF-7 cells with a lentiviral vector encoding either of two independent shRNAs, which targeted different nucleotide sequences in ZNF217 mRNA, or a non-targeting control shRNA (NTC). Knockdown of ZNF217 significantly increased levels of total m^6^A^+^ RNA (Figure 4B), m^6^A^+^ NANOG mRNA (Figure 4C), and m^6^A^+^ KLF mRNA (Figure 4D) under both non-hypoxic and hypoxic conditions. In addition, knockdown of ZNF217 inhibited the induction of NANOG (Figure 4E) and KLF4 (Figure 4F) mRNA expression under hypoxic conditions. Two-way ANOVA demonstrated interaction between the two variables (i.e. hypoxia and ZNF217 knockdown). Compared to the NTC subclone, NANOG and KLF4 protein levels were also decreased in the ZNF217 knockdown subclones (Figure 4G).

We previously demonstrated that ALKBH5 knockdown increased the levels of m^6^A^+^ NANOG mRNA and decreased the hypoxic induction of NANOG mRNA expression [13]. We next tested whether m^6^A^+^ KLF4 mRNA was also demethylated by ALKBH5. Similar to ZNF217 knockdown, ALKBH5 knockdown led to increased m^6^A^+^ KLF4 mRNA in MCF-7 cells under both normoxic and hypoxic conditions (Figure 4H). The hypoxic induction of KLF4 mRNA (Figure 4I) and protein (Figure 4J) expression was inhibited in ALKBH5 knockdown subclones. Taken together with our previous studies [13], the data in Figure 4 indicate that ZNF217 and ALKBH5 positively regulate NANOG and KLF4 expression by decreasing m^6^A methylation of NANOG and KLF4 mRNA, particularly under hypoxic conditions.

### ZNF217 deficiency impairs hypoxia-induced enrichment of BCSCs

**Figure 5:**
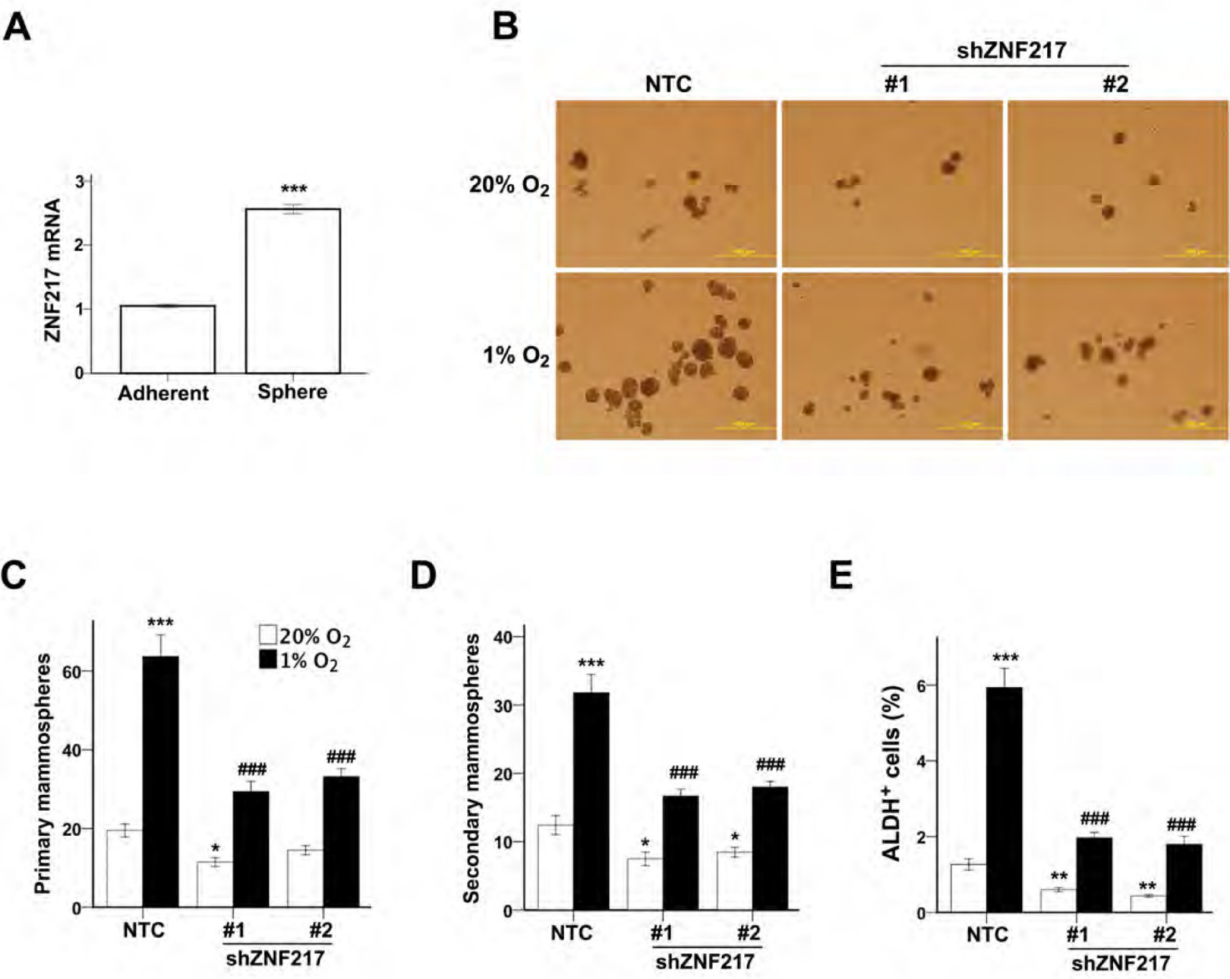
ZNF217 is required for hypoxia-induced BCSC enrichment **A.** ZNF217 mRNA levels were determined in MCF-7 parental cells, which were cultured as adherent monolayers or as mammospheres (mean ± SEM; *n*= 3). ***P < 0.001 *vs*. adherent cells by Student's *t* test. **B**-**D.** Monolayer cultures of MCF-7 subclones were exposed to 20% or 1% O_2_ for 72 h and transferred to ultra-low attachment plates. The number of primary (B and C) and secondary (D) mammospheres per 1,000 cells initially seeded was determined (mean ± SEM; *n* = 3). *P < 0.05, ***P < 0.001 *vs*. NTC at 20% O_2_; ###*P* < 0.001, *vs*. NTC at 1% O_2_ by two-way ANOVA. Scale bar: 500 μm. **E**. MCF-7 subclones were exposed to 20% or 1% O_2_ for 72 h and the percentage of ALDH+ cells was determined (mean ± SEM; *n* = 3). **P < 0.01, ***P < 0.001 *vs*. NTC at 20% O_2_; ###*P* < 0.001 *vs*. NTC at 1% O_2_ by two-way ANOVA.

To investigate whether ZNF217 regulates the BCSC phenotype, we first performed RT-qPCR to measure ZNF217 mRNA levels in parental MCF-7 cells grown as adherent monolayers or as non-adherent mammospheres, which are enriched for BCSCs [29]. ZNF217 mRNA levels were significantly higher in mammospheres as compared to adherent cells (Figure 5A). Next, we analyzed the effect of ZNF217 depletion on the specification/maintenance of BCSCs. NTC and ZNF217 knockdown subclones were exposed to 20% or 1% O_2_ for 72 h, transferred to ultra-low adherence plates, incubated at 20% O_2_ for one week, and the number of primary mammospheres were counted (Figure 5B). The primary mammospheres were then dissociated, the cells were replated on ultra-low adherence plates, incubated at 20% O_2_ for another week, and the number of secondary mammospheres were counted, which provides a more stringent analysis of self-renewal capability. Exposure of the NTC subclone to hypoxia significantly increased the number of primary (Figure 5C) and secondary (Figure 5D) mammospheres, indicating that hypoxia induces the BCSC phenotype, which is maintained for at least two weeks under non-hypoxic conditions, as previously described [17]. These results suggest that cells exposed to hypoxia in the primary tumor may maintain the BCSC phenotype even after they have entered the circulation and arrested in a non-hypoxic site of metastasis such as the lungs. The number of primary and secondary mammospheres was significantly decreased in the ZNF217 knockdown subclones exposed to 20% or 1% O_2_ for 3 days prior to the mammosphere assays (Figure 5B-D). Consistent with the mammosphere assays, the percentage of ALDH^+^ cells in ZNF217 knockdown subclones was also significantly decreased under both non-hypoxic and hypoxic conditions as compared to the NTC subclone (Figure 5E). Two-way ANOVA demonstrated interaction between the two variables (i.e. hypoxia and ZNF217 knockdown). Taken together, the data in Figure 5 indicate that, as in the case of ALKBH5, ZNF217 is a positive regulator of the BCSC phenotype.

### ALKBH5 expression is correlated with HIF-1α expression in human breast cancer biopsies

**Figure 6:**
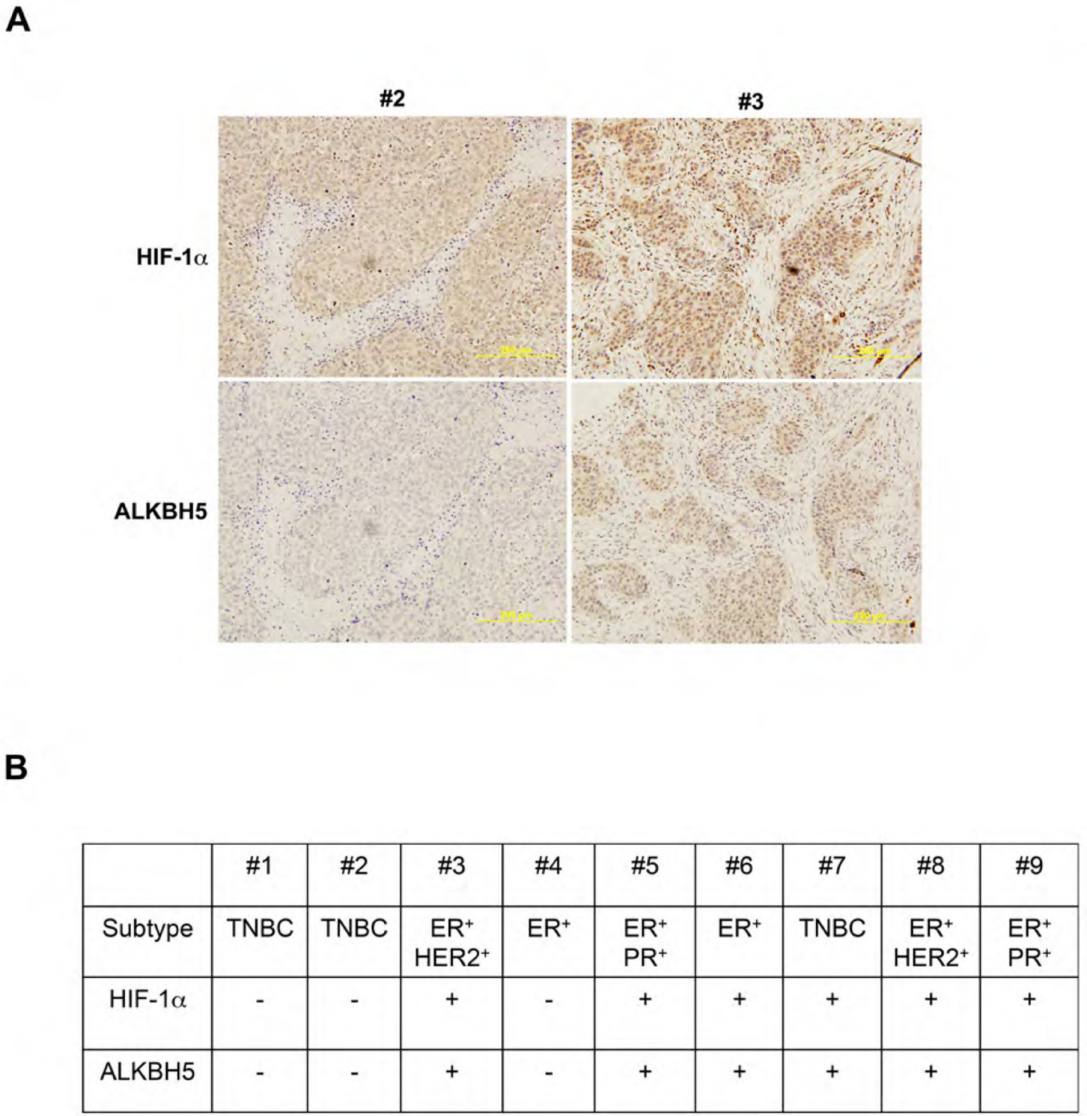
Immunohistochemical analysis of human breast cancer biopsies **A.** Immunohistochemistry was performed on breast cancer biopsies using ALKBH5 and HIF-1α antibodies. Representative negative and positive staining (biopsies from patients #2 and #3, respectively) are shown. Scale bar: 500 μm. **B.** Summary of ALKBH5 and HIF-1α expression in nine human breast cancers of the indicated subtypes based on expression of ER, PR, and HER2. TNBC, triple negative breast cancer.

We next analyzed the expression of ALKBH5 and HIF-1α in human breast cancer biopsies. Available antibodies against ZNF217 were not of suitable quality for immunohistochemistry. Because hypoxia-induced ALKBH5 expression is HIF-dependent in breast cancer cell lines [13, 14] and human breast cancers are hypoxic, with a median pO_2_ of 10 mm Hg (∼1.5% O_2_) [30], we hypothesized that ALKBH5 and HIF-1α expression should be correlated in breast cancer tissue. We performed immunohistochemistry on sections from 9 representative breast cancer biopsies to analyze ALKBH5 and HIF-1α expression. Sections with > 5% stained cells were classified as positive for ALKBH5 or HIF-1α. Three biopsies were negative for both ALKBH5 and HIF-1α (including #2 in Figure 6A). Six biopsies were positive for both ALKBH5 and HIF-1α (including #3 in Figure 6A). Thus, ALKBH5 and HIF-1α expression were concordant in all 9 breast-cancer biopsies analyzed (Figure 6B).

### ALKBH5 expression is required for efficient tumorigenesis and lung metastasis

**Figure 7:**
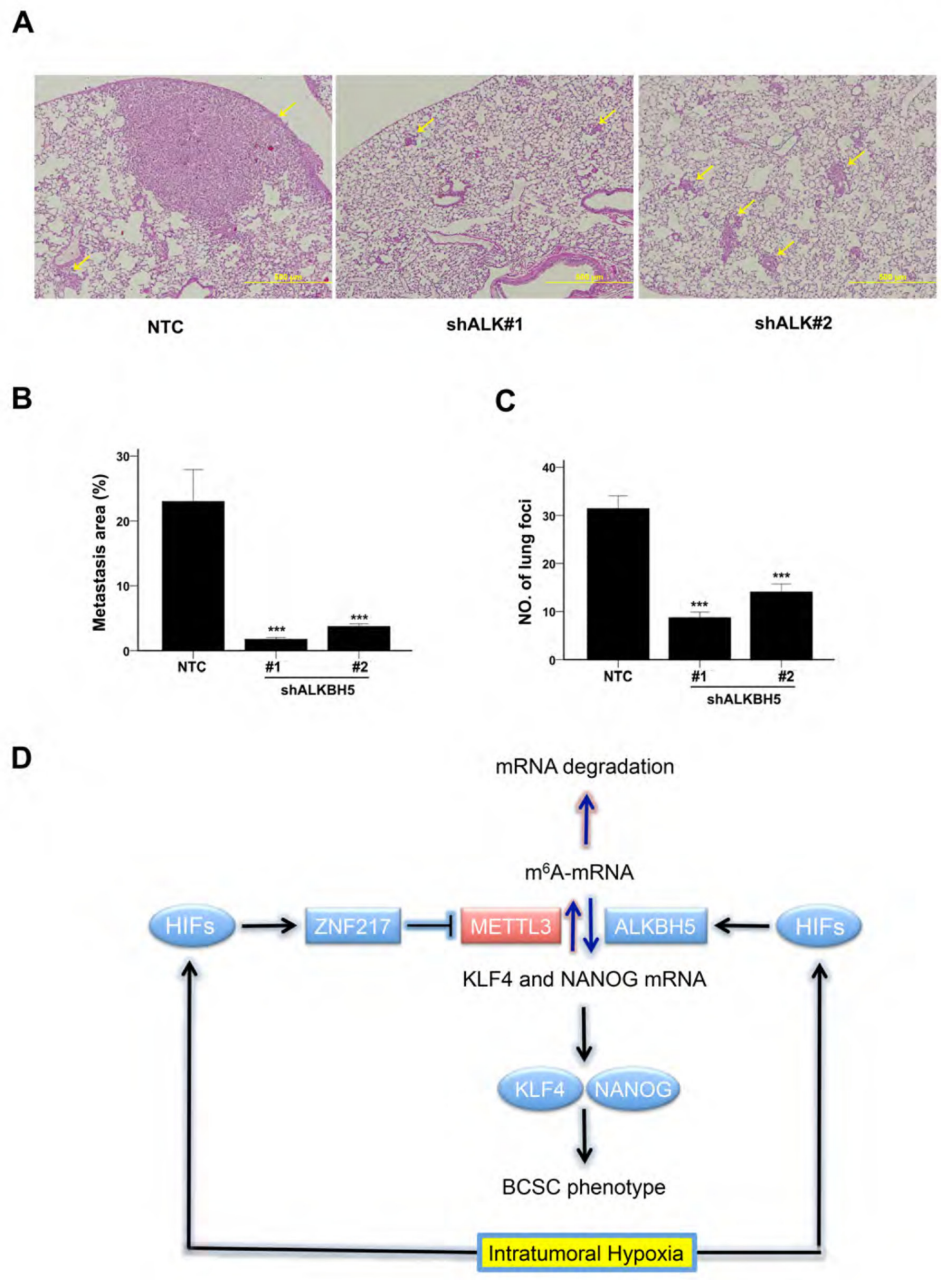
ALKBH5 is required for breast cancer tumorigenicity and metastasis **A-C.** Analysis of lung metastasis after orthotopic transplantation of MDA-MB-231 subclones in female SCID mice. Representative hematoxylin-and-eosin stained sections showing lung metastases (arrows) in mice that received mammary fat pad injections of NTC or shALKBH5 cells; scale bar: 500 μm (A). Image analysis was performed and the percentage of lung area occupied by metastases (B) and the number of metastatic foci per lung section (C) were determined (mean + SEM, *n* = 3). ***P < 0.001 *vs*. NTC by ANOVA. **D.** Hypoxia induces HIF-dependent expression of ZNF217 and/or ALKBH5. ZNF217 interacts with METTL3 and inhibits METTL3-catalyzed m6A methylation of KLF4 and NANOG mRNA, whereas ALKBH5 demethylates m6A+ mRNA. m6A-free KLF4 and NANOG mRNAs are stabilized, leading to increased levels of KLF4 and NANOG protein, which specify the BCSC phenotype.

Because ZNF217 functions as a transcriptional regulator in addition to its role as an inhibitor of m^6^A methylation [23], whereas the only known function of ALKBH5 is to demethylate m^6^A residues in RNA, we decided to study the effect of ALKBH5 loss of function *in vivo*, using orthotopic transplantation of the triple-negative breast cancer cell line MDA-MB-231. 1×10^3^ cells of NTC and shALKBH5 subclones were injected into the mammary fat pad of female SCID mice. Ten weeks after injection, 100% (7/7) of the mice that were injected with NTC cells developed palpable tumors, as compared to only 43% (6/14) of the mice injected with ALKBH5-deficient cells, as previously reported [13]. When the primary tumor volume reached 1000 mm^3^, the lungs were fixed under inflation and paraffin sections were stained with hematoxylin and eosin to identify metastatic foci (Figure 7A). Image analysis was performed to calculate the percentage of lung area occupied by metastases (Figure 7B) and the number of metastatic foci (Figure 7C). Whereas mice injected with NTC cells formed many large metastases, only a few small collections of metastatic cells were observed in the lungs of mice injected with ALKBH5 knockdown cells.

## DISCUSSION

Intratumoral hypoxia, caused by dysregulated cell proliferation in combination with abnormal blood vessel formation and function, is one of the critical features of the tumor microenvironment that drives cancer progression [30-32]. In this context, HIFs are overexpressed in many advanced cancers, including breast cancer, and activate the transcription of large batteries of genes that are required for metastasis [33]. Recent studies have demonstrated that HIFs are required for the specification and/or maintenance of BCSCs in response to hypoxia or chemotherapy by activating multiple signaling pathways [7, 8, 13, 16, 17, 26, 34-37]. Gene expression microarray analyses have revealed the remarkable molecular heterogeneity of breast cancers [38]. In the present study, hypoxia induced the HIF-dependent enrichment of BCSCs in all breast cancer cell lines, which was accompanied by increased expression of one or more pluripotency factors (NANOG, KLF4, or SOX2). The heterogeneous expression of pluripotency factors in response to hypoxia was, in turn, associated with heterogeneity with respect to the induction of ZNF217 and ALKBH5, which was also HIF-dependent (Figure 7D). ZNF217 and ALKBH5 play complementary roles in negatively regulating m^6^A levels in RNA: ZNF217 inhibits methylation, whereas ALKBH5 induces demethylation. The hypoxia-induced expression of ZNF217 or ALKBH5 was required for: m^6^A demethylation of KLF4 or NANOG mRNA; increased levels of KLF4 or NANOG mRNA and protein; and enrichment of BCSCs. SOX2 mRNA was also subject to m^6^A methylation in ES cells [39]. Depletion of Zfp217, the mouse homolog of ZNF217, in ES cells, increased m^6^A modification of NANOG, KLF4, and SOX2 mRNAs, promoting their degradation [23]. This suggests that the observed hypoxia-induced expression of SOX2 in ZR75.1 cells may reflect increased expression of ZNF217 (Table 1), leading to decreased m^6^A modification of SOX2 mRNA. Remarkably, in mouse ES cells, Zfp217 bound directly to the *Nanog* and *Sox2* genes and activated their transcription [23], suggesting that ZNF217 may regulate the expression of pluripotency factors by both transcriptional and post-transcriptional mechanisms.

Although all seven breast cancer cell lines displayed BCSC enrichment and increased expression of one or more pluripotency factors in response to hypoxia, neither ALKBH5 nor ZNF217 expression was induced by hypoxia in SUM149 or T47D cells (Table 1). Thus, there appears to be yet another molecular mechanism that leads to increased expression of SOX2 in T47D and KLF4 in SUM149 cells. One possibility is that HIFs directly activate *SOX2* and *KLF4* gene transcription. Exposure of human ES cells to hypoxia induced the binding of HIF-2α to the *NANOG* and *SOX2* promoters [40]. Taken together with our results, these data suggest that HIFs may increase the expression of pluripotency factors either by a direct, transcriptional mechanism or by an indirect, post-transcriptional mechanism in which ALKBH5 and ZNF217 mediate decreased m^6^A modification leading to increased stability of NANOG and KLF4 mRNA.

Several studies have demonstrated that ZNF217 promotes breast cancer progression. Overexpression of ZNF217 promoted mammosphere formation and increased metastasis in an orthotopic mouse model of breast cancer [20]. High expression of ZNF217 is correlated with increased breast cancer mortality [20, 22]. In our studies, loss of ALKBH5 expression significantly reduced the tumorigenicity of MDA-MB-231 cells and, in tumor-bearing mice, metastatic burden in the lungs was also significantly decreased. Immunohistochemical analysis revealed that HIF-1α and ALKBH5 expression were highly correlated in human breast cancer biopsies, suggesting that the data from *in vitro* and *in vivo* analysis of breast cancer cell lines are clinically relevant.

HIFs function as master regulators by activating the transcription of multiple genes within a biological pathway [41]. For example, hypoxia promotes breast cancer cell motility by inducing the expression of the genes encoding RhoA and RhoA kinase [42]. HIFs promote premetastatic niche formation by regulating the expression of multiple members of the LOX family of proteins (LOX, LOXL2, LOXL4) and different family members were found to be expressed in different human breast cancer biopsies and induced by hypoxia in different breast cancer cell lines, but in all cases the induction was HIF-dependent and could be blocked by HIF inhibitors [27, 43]. Similarly, different pluripotency factors were induced by hypoxia as a result of increased expression of ALKBH5 or ZNF217, but in all cases, the induction was HIF-dependent. Our results illustrate how different cancers can reach the same pathophysiological endpoint by utilization of different signaling pathways that are controlled by a single master regulator. HIFs represent a rational target for breast cancer therapy because of the essential and pleiotropic role that they play in BCSC specification [7, 8, 13, 16, 17, 26, 34-37]; resistance to endocrine therapy [44-46] or chemotherapy [7, 8]; and distant metastasis [33], which together constitute the lethal cancer phenotype.

## MATERIALS AND METHODS

### Cell culture

Cell authentication and mycoplasma testing were performed at the Johns Hopkins Genetics Core Resources Facility by PCR analysis. MCF-7 and MDA-MB-231 cells were cultured in Dulbecco's modified Eagle medium (DMEM). T47D, HCC-1954, and ZR75.1 cells were cultured in RPMI-1640. SUM-149 and SUM-159 cells were cultured in DMEM/F12 (50:50) supplemented with hydrocortisone and insulin. All culture media were supplemented with 10% (vol/vol) fetal bovine serum and 1% (vol/vol) penicillin/streptomycin. Cells were maintained in a 5% CO_2_ and 95% air incubator (20% O_2_). For hypoxia exposure, cells were placed in a modular incubator chamber, which was flushed for 2 min at 2 psi with a gas mixture containing 1% O_2_, 5% CO_2_, and 94% N_2_.

### RT-qPCR

Total RNA was extracted using TRIzol (ThermoFisher Scientific, Waltham, MA) according to the manufacturer's instructions. cDNA synthesis was performed using the High Capacity RNA-to-cDNA Kit (Applied Biosystems, Foster City, CA). qPCR was performed using following primers: NANOG, 5′-TTT GTG GGC CTG AAG AAA ACT-3′ and 5′-AGG GCT GTC CTG AAT AAG CAG-3′; KLF4, 5′-CGG ACA TCA ACG ACG TGA G-3′ and 5′-GAC GCC TTC AGC ACG AAC T-3′; SOX2, 5′- GCC GAG TGG AAA CTT TTG TCG-3′ and 5′-GGC AGC GTG TAC TTA TCC TTC T-3′; ZNF217 5′-AAA CAT GCC AAC TCA ATC CCT C-3′ and 5′-GGA ATG GAA CAA CAG CGG T-3′; FTO, 5′-GCT GCT TAT TTC GGG ACC TG-3′ and 5′-AGC CTG GAT TAC CAA TGA GGA-3′; and 18S rRNA, 5′-CGG CGA CGA CCC ATT CGA AC-3′ and 5′-GAA TCG AAC CCT GAT TCC CCG TC-3′. The expression (E), in cells exposed to 20% or 1% O_2_, of each target mRNA, relative to 18S rRNA, was calculated based on the cycle threshold (Ct) using the formula E = 2e-∆(∆Ct) in which ∆Ct = Ct_target_ - Ct_18S_ and ∆(∆Ct) = ∆Ct_20%_ - ∆Ct_1%_. Mean ± SEM are reported, based on 3 technical replicates for each of 3 biological replicates.

### Immunoblot assay

Whole cell lysates were prepared in modified RIPA lysis buffer (50 mM Tris-HCl [pH 7.5], 1 mM β-mercaptoethanol, 150 mM NaCl, 1 mM Na_3_VO_4_, 1 mM NaF, 1 mM EDTA, 0.25% sodium deoxycholate and 1% Igepal CA-630). Blots were probed with HIF-1α (#610959, BD Biosciences, San Jose, CA), HIF-2α (#NB100-122, Novus Biologicals, Littleton, CO), ALKBH5 (#NBP1-82188, Novus Biologicals), NANOG (#NB100-588, Novus Biologicals), KLF4 (#NBP1-83940, Novus Biologicals), SOX2 (NBP1-42823, Novus Biologicals), ZNF217 (#NBP1-78189, Novus Biologicals), and Actin (#sc-1616, Santa Cruz Biotechnology, Dallas, TX) antibodies. HRP-conjugated anti-rabbit and anti-mouse secondary antibodies were used and the chemiluminescent signal was detected using ECL Plus (GE Healthcare Life Sciences, Marlborough, MA). Three different breast cancer cell lines or 3 biological replicates of a single cell line were analyzed.

### Lentiviral vectors and transduction

Lentiviral vectors encoding shRNA targeting HIF-1α and HIF-2α were described previously [28]. pLKO.1-puro lentiviral vectors encoding shRNA targeting ALKBH5 mRNA (clone ID: NM_017758.2-1625s1c1 and NM_017758.2-1176s1c1) and ZNF217 (clone ID: NM_006526.2-2363s1c1 and NM_006526.2-2951s21c1) were purchased from Sigma-Aldrich (St. Louis, MO). All lentiviral vectors were transfected into 293T cells for packaging. Viral supernatant was collected after 48 h and used for transfection. Puromycin (0.5 μg/ml) was added to the medium of cells transduced with lentivirus for selection.

### Measurement of m^6^A in total RNA and m^6^A^+^ NANOG and KLF4 mRNA levels

Total m^6^A content was measured in 200-ng aliquots of total RNA extracted from MCF-7 subclones using an m^6^A RNA methylation quantification kit (#P-9005, Epigentek, Farmingdale, NY) according to the manufacturer's instructions. For measurement of m^6^A^+^ NANOG and KLF4 mRNA levels, RNA immunoprecipitation using an m^6^A antibody (#202003, Synaptic Systems, Atlanta, GA) was performed as previously described [13, 47]. RT-qPCR was performed to amplify the region containing an m^6^A consensus sequence within NANOG and KLF4 mRNA using the following primers: NANOG, 5′-ATG CAA CCT GAA GAC GTG TG-3′ and 5′-GAG ATT GAC TGG ATG GGC AT-3′; and KLF4, 5′-ACC TGC GAA CCC ACA CAG-3′ and 5′-GGT GCC CCG TGT GTT TAC-3′. Mean ± SEM are reported, based on 3 biological replicates.

### Mammosphere assay

After exposure to 20% or 1% O_2_ for 72 h, MCF-7 cells were trypsinized and seeded in six-well ultra-low attachment plates (Corning, Corning, NY) at a density of 10,000 cells per ml in Complete MammoCult Medium (StemCell Technologies, Vancouver, BC). After 7 d, the cells were photographed under a TH4-100 microscope (Olympus, Center Valley, PA) and primary mammospheres with diameter ≥ 70 μm were counted. For secondary mammosphere formation, primary mammospheres were trypsinized, plated at a density of 10,000 cells per ml, incubated for 7 d and counted. Mean ± SEM are reported, based on 3 biological replicates.

### ALDH assay

MCF-7 cells were exposed to 20% or 1% O_2_ for 72 h and harvested for Aldefluor assay (StemCell Technologies). Single-cell suspensions (5 × 10^5^ cells in 1 ml of assay buffer) were incubated with BODIPY-aminoacetaldehyde (1 μM) at 37°C for 45 min, followed by flow cytometry. Mean ± SEM are reported, based on 3 biological replicates.

### Immunohistochemistry

Formalin-fixed and paraffin-embedded sections (4-μm thick) from 9 human breast cancer biopsies were deparaffinized and rehydrated, followed by antigen retrieval using citrate buffer (pH 6.1). Staining was performed using HIF-1α (BD Biosciences) or ALKBH5 (Novus Biologicals) antibody and the LSAB+ System HRP kit (DAKO, Carpinteria, CA) according to the manufacturer's instructions.

### Orthotopic transplantation and analysis of lung metastasis

Protocols were approved by the Johns Hopkins University Animal Care and Use Committee and were in accordance with NIH guidelines [48]. 1,000 MDA-MB-231 subclone cells were injected into the mammary fat pad of randomly chosen 6-to-8 week-old female severe combined immunodeficiency (SCID) mice in a 1:1 suspension of Matrigel (BD Biosciences) in PBS (n = 7 mice per subclone). 10 weeks after injection, mice were examined for the presence of primary tumors [13]. When primary tumors reached a volume of 1000 mm^3^, the lungs were perfused with PBS and inflated for formalin fixation and paraffin embedding. Sections (4-μm thick) were stained with hematoxylin and eosin. For metastasis quantification, 3 random fields were analyzed per section and 3 sections were analyzed per mouse using Image J software (NIH) by a blinded investigator. The mean value of the number of metastatic foci and the percentage of lung area occupied by metastases in each group was calculated (mean ± SEM).

### Statistical analysis

Differences between two groups were analyzed by Student's t test. Differences between more than two groups were analyzed by two-way ANOVA. For each assay, variance was similar between groups.
